# Plasma ADAMTS13 and von Willebrand factor in diagnosis and prediction of prognosis in pulmonary arterial hypertension

**DOI:** 10.1177/20458940211041500

**Published:** 2021-09-30

**Authors:** Abdulla Ahmed, Salaheldin Ahmed, Göran Rådegran

**Affiliations:** 1 Department of Clinical Sciences Lund, The Section for Cardiology, 5193 Lund University, Lund, Sweden; 2 The Haemodynamic Lab, The Section for Heart Failure and Valvular Disease, Heart and Lung Medicine, Skåne University Hospital, Lund, Sweden

**Keywords:** Biomarkers, diagnosis, pulmonary arterial hypertension, prognosis

## Abstract

To improve outcome in pulmonary arterial hypertension, earlier diagnosis and better prognostic assessments are required. We aimed to investigate the diagnostic and prognostic potential of plasma proteins related to pathways recognized in pulmonary arterial hypertension including coagulation, inflammation, and metabolism. Forty-two proteins were analysed with proximity extension assay from plasma of 20 healthy controls and 150 patients, including (pulmonary arterial hypertension, n = 48, whereof 33 also during early treatment follow-ups); chronic thromboembolic pulmonary hypertension (CTEPH, n = 20); pulmonary hypertension (PH) due to heart failure (HF) with preserved ejection fraction (HFpEF-PH, n = 31); PH due to HF with reduced ejection fraction (HFrEF-PH, n = 36); and HF without PH (Dyspnoea/HF-non-PH, n = 15). Patients’ haemodynamics were assessed by right heart catheterization. Plasma ADAMTS13 in incident pulmonary arterial hypertension was lower compared to the healthy controls (p = 0.055), as well as CTEPH (p < 0.0001), HFrEF-PH (p < 0.0001), HFrEF-PH (p < 0.0001), and Dyspnoea/HF-non-PH (p < 0.0001). Adjusted for age and sex, ADAMTS13 discriminated pulmonary arterial hypertension from the other disease groups with an AUC of 0.91 (sensitivity = 87.5%, and specificity = 78.4%). Higher plasma von Willebrand factor was associated with worse survival (log-rank p = 0.0029), and a higher mortality rate (adjusted hazard ratio 1.002, 95% confidence interval 1–1.004; p = 0.041). Adjusted for age, sex, and combined with the ESC/ERS risk score, von Willebrand factor predicted mortality (median follow-up 3.6 years) in pulmonary arterial hypertension with an AUC of 0.94 (sensitivity = 81.3%, and specificity=93.8%). ADAMTS13 may be a promising biomarker for early detection of PAH and von Willebrand factor as a candidate prognostic biomarker. The putative additional value of von Willebrand factor to the European multiparametric risk assessment strategy remains to be elucidated.

## Introduction

Pulmonary hypertension (PH) imposes a major health issue, affecting approximately 1% of the population globally and up to 10% of individuals aged >65 years,^1^ with impact on morbidity and mortality.^2^ Pulmonary arterial hypertension (PAH, WHO group I) is a rare form of PH, considered as an angioproliferative vasculopathy,^1^ in which endothelial dysfunction with excessive vasoconstriction, as well as remodelling of the pulmonary arterioles ensue, leading to a raise in the pulmonary arterial pressure and pulmonary vascular resistance. As an adverse corollary, an increase in right ventricular afterload emerges, resulting in progressive right heart failure, syncope, and premature death.^13–6^

In PAH, pulmonary vascular endothelial dysfunction contributes to vascular remodelling.^7^ Endothelial dysfunction is distinguished by impairment of the endothelium-dependent vasodilatation favouring vasoconstriction, but also encompasses metabolic derangement, local release of cytokines, and reduced anticoagulant properties.^3^ For instance, excessive local secretion of interleukin-1, interleukin-6, and tumour necrosis factor-α contribute, among other inflammatory mediators, to an integral part in the structural and functional vascular alterations in PAH.^37^ Additionally, an imbalance in vasoactive mediators including nitric oxide and prostacyclin, both of which are decreased in patients with PAH, inhibit platelet aggregation.^8^

Apart from the evident perturbations of molecular mechanisms in PAH,^8^ a major clinical issue is the diagnostic delay in patients with PAH. In a recent study, the median time from symptom onset to diagnostic right heart catheterization (RHC) was 1.2 years, which is not different from the median diagnostic delay reported in the 1980s.^910^ In addition, the mean diagnostic delay is reported to be ∼2.5 years, where 35% of the patients had a diagnostic delay of >2 years.^9^ Importantly, a delay exceeding two years in PAH patients, irrespective of age, sex, and the associated PAH subgroup, is associated with an 11% increase in mortality rates,^910^ underlying that early diagnosis and treatment initiation are paramount to achieve better outcomes.^9^

Clinical management of PAH involves assessment of disease severity.^41112^ The current European Society of Cardiology and the European Respiratory Society (ESC/ERS) guidelines in assessing prognosis are based on a multidimensional approach involving clinical assessment, biochemistry, and imaging.^11^ However, the prognostic accuracy, based on data from the Registry to Evaluate Early and Long-Term PAH Disease Management (REVEAL), of the established risk assessment instruments are still lacking with concordance statistics ranging from 0.62 for the Comparative, Prospective Registry of Newly Initiated Therapies for Pulmonary Hypertension (COMPERA),^13^ 0.64 for the French Pulmonary hypertension Registry (FPHR),^14^ to 0.76 for the REVEAL 2.0,^15^ allowing for considerable improvement.

In the clinical context, biomarkers, including proteomics,^16^ for screening of individuals with symptoms of dyspnoea or at-risk of developing right heart failure, may provide a promising means to reduce the ‘great diagnostic delay’ experienced by patients with PAH. In addition, identifying prognostic biomarkers, reflecting the pathways involved in the development and progression of PAH, may be of great value for further refinement of risk assessment. Thus, in search of biomarker candidates with the potential of decreasing the diagnostic delay and improve existing PAH risk assessment, we aimed to investigate the diagnostic and prognostic potential of several plasma proteins, involved in coagulation, inflammation, and metabolism, in PAH.

## Materials and methods

### Study population

Between October 2011 and April 2017, 155 patients evaluated at the Haemodynamic lab at Skåne University Hospital in Lund, as well as 20 young, healthy controls were enrolled in Lund Cardio Pulmonary Registry (LCPR). LCPR is a prospective cohort of Region Skåne’s biobank biobank comprising blood samples and clinical data. All participants were ≥18 years upon enrollment. Patients with missing haemodynamic data (n = 3) or exhibiting comorbidities imposing a direct influence on the definite diagnosis (n = 2) were excluded. The present study included all eligible patients enrolled in LCPR. All participants provided written informed consent and the study conforms with the ethical standards outlined in the declaration of Helsinki and Istanbul. The study was approved by the regional ethical board in Lund, Sweden (Dnr: 2010/114, 2010/442, 2011/368, 2011/777, 2014/92, 2015/270).

### Blood samples and proteomic analysis

The participants were not fasting, and venous blood was sampled from patients at the time of RHC, whereas venous blood from controls was sampled at routine clinical examinations. Mixed venous samples were drawn during the RHC from the introducer in vena jugularis interna. This sampling method was applied on all patients included in the present work. As for the healthy controls, samples were taken peripherally via vena mediana cubiti. Blood samples were collected using 6 mL EDTA BD Vacutainer tubes, centrifuged at 2000 r/min × 10 min, after sampling. Plasma aliquots were stored in LCPR at –80°C until required.

In the present study, plasma samples were analyzed from incident (newly diagnosed), treatment-naïve PAH (WHO group I) patients (n = 48), as well as patients with chronic thromboembolic PH (CTEPH, n = 20, WHO group IV), PH due to HF (WHO group II) with preserved ejection fraction (HFpEF-PH, n = 31), PH due to HF with reduced EF (HFrEF-PH, n = 36), as well as a HF population consisting of eight patients with HFrEF and seven with HFpEF, regarded as a dyspnoea non-PH control group (Dyspnoea/HF-non-PH). Additionally, samples from a subset of the PAH population during an early first treatment follow-up (n = 33), as well as 20 healthy controls were also analyzed.

Forty-two selected plasma proteins reflecting several pathways recognized in the pathophysiology of PAH, including coagulation, metabolism, and inflammation,^34^ in addition to N-terminal pro-brain natriuretic peptide (NT-proBNP), were analyzed with proximity extension assay (PEA), using multiplex immunoassay reagent kits (cardiovascular II, III, and oncology II panels). The plasma proteins included alpha-L-iduronidase (IDUA), a disintegrin and metalloproteinase with thrombospondin motifs 13 (ADAMTS13), agouti-related protein (AGRP), bone morphogenetic protein 6 (BMP-6), 9 (BMP-9), carbonic anhydrase VA (CA-VA), chymotrypsin-C, delta-like protein 1 (DLL1), Dickkopf-related protein 1 (Dkk-1), follistatin, gastric intrinsic factor, heat shock protein beta-1 (HspB1), heme oxygenase 1, hydroxyacid oxidase 1 (HAOX1), mothers against decapentaplegic homolog 5 (Mad homolog 5), neurogenic locus notch homolog protein 3 (Notch 3), NF-kappa-B essential modulator (NEMO), pancreatic prohormone (PPY), plasminogen activator inhibitor 1 (PAI-1), poly [ADP-ribose] polymerase 1 (PARP-1), programmed cell death 1 ligand 2 (PD-L2), proteinase-activated receptor 1 (PAR-1), protein delta homolog 1 (DLK-1), r-spondin-3, seizure 6-like protein (SEZ6L), serine protease 27 (PRSS27), serine/threonine-protein kinase 4 (MST-1), somatotropin, spondin-1, spondin-2, superoxide dismutase [Mn] mitochondrial (SOD2), TGF-beta receptor type-2 (TGFBRII), thrombomodulin, thrombopoietin, tissue factor, tissue factor pathway inhibitor (TFPI), 2 (TFPI-2), tissue-type plasminogen activator (t-PA), urokinase plasminogen activator surface receptor (uPAR), urokinase-type plasminogen activator (uPA), von Willebrand factor (vWF), and Wnt inhibitory factor 1 (WIF-1).

The PEA technique is based on DNA oligonucleotide-labelled antibodies. Upon pairwise binding of the oligonucleotide-labelled antibodies to the target antigen, the oligonucleotides come into proximity, hybridize, and are extended by a DNA-polymerase. Subsequently, the DNA template is quantified by microfluidic qPCR (Biomark HD, Fluidigm, San Francisco, CA, USA).^17^ Each panel correspond to a plate on which the multiplex analysis was performed. In each plate, inter-plate controls were added in triplicates, and the median of the inter-plate triplicates was used to adjust for inter-plate variations. Moreover, the panels are validated for sensitivity, specificity, dynamic range, precision, and scalability (Olink proteomics, Uppsala, Sweden).^17^ By default, the proteins’ levels of the assays from Olink are expressed on an arbitrary log_2_ scale (AU-log2), reflecting the inverted Ct values. In the present study, as the log_2_ scale did not provide normally distributed data, the logarithmic values were transformed to a linear protein expression scale (AU), using the following formula: linear AU = 2^AU-log^^2^.

### RHC and haemodynamics

RHC was performed in the supine position at rest, predominantly via the right internal jugular vein, using a Swan Ganz catheter (Baxter Health Care Corp, Santa Ana, CA). Mean arterial pressure (mAP), systolic pulmonary arterial pressure (sPAP), diastolic pulmonary arterial pressure (dPAP), pulmonary arterial wedge pressure (PAWP), and mean right atrial pressure (mRAP) were measured during the RHCs. In addition, cardiac output (CO) was measured by thermodilution and heart rate by ECG. Cardiac index (CI), stroke volume (SV), SV index (SVI), transpulmonary pressure gradient (TPG), pulmonary arterial compliance (PAC), pulmonary vascular resistance (PVR), left and right ventricular stroke work indices (LVSWI and RVSWI) were calculated using the following formulae: CI = CO/body surface area (BSA), SVI = CI/HR, TPG = mPAP–PAWP, PVR = TPG/CO, PAC = SV/(sPAP–dPAP), LVSWI = (mAP–PAWP) × SVI and RVSWI = (mPAP–mRAP) × SVI.

### Diagnostic procedures

The patients were diagnosed by experienced cardiologists. HF was classified using echocardiography and magnetic resonance imaging. HFpEF and HFrEF were defined as EF ≥50% and <50%, respectively. PH due to left heart disease was defined as a resting mPAP ≥25 mmHg at a PAWP >15 mmHg, except for five patients exhibiting a borderline PAWP of 15 mmHg. PAH was defined in accordance with prevailing ESC/ERS guidelines, including a resting mPAP ≥25 mmHg, PAWP ≤15 mmHg and PVR >3 wood units (WU).^11121821^ The PH centre at Skåne University Hospital constitutes one of the data sources to the SPAHR registry. Echocardiography and magnetic resonance imaging were performed to identify intracardiac shunts. CTEPH was identified with pulmonary scintigraphy. High-resolution computed tomography and spirometry with diffusion capacity were performed for assessment of lung disease including interstitial lung disease, pulmonary fibrosis, and emphysema, to exclude patients with PH due to hypoxia and/or lung disease (WHO group III PH). Our cohort included PAH patients with systemic sclerosis (SSc), where none exhibited a picture concordant with WHO group III PH. All patients underwent echocardiography assessment and provocative testing if needed including fluid challenge to rule out occult diastolic dysfunction (WHO group II PH).

### Risk assessment

World Health Organization functional class, six-minute walking distance, NT-proBNP, mRAP, CI, and mixed venous oxygen saturation (SvO_2_) were used to calculate the risk scores of the PAH patients at baseline, using the calculation method described by Kylhammar et al.,^12^ previously utilized to validate the risk assessment instrument from the 2015 ESC/ERS guidelines.^11^ Each of the patients’ parameters were graded with a score of 1 for low risk, 2 for intermediate risk, and 3 for high risk. The graded scores were in accordance with the ESC/ERS provided cut-offs. The mean of the available variables is subsequently rounded off to the nearest integer, rendering a risk score.^12^ In the present study, to retain more detailed data, the means were expressed with two decimals.

### Renal function

The creatinine-based estimates of the patients’ glomerular filtration rate (eGFR) were calculated using the revised Lund-Malmö equation. Included variables in the eGFR equation in addition to creatinine were age and sex.^22^

### Statistics

Statistical analyses were performed using R version 4.0.2 (R Foundation for Statistical Computing, Vienna, Austria) and GraphPad Prism version 9.00 (GraphPad Software, La Jolla, CA, USA, www.graphpad.com). Data distribution was determined by histograms. Unless otherwise stated, data were presented as medians (25th–75th percentiles; interquartile range (IQR)). Mann-Whitney U and Wilcoxon matched-pairs signed-rank tests were performed as appropriate. When comparing multiple groups, Kruskal-Wallis tests were performed followed by multiple comparisons. Survival was estimated using the Kaplan-Meier method, and difference between groups was determined by the log-rank test. Correlations were expressed by Spearman’s rank coefficient (r_s_). To accommodate for mass significance, the two-stage step-up method of Benjamini, Krieger, and Yekutieli was used to calculate the false discovery rate, (FDR) set at (Q = 0.05) for healthy controls versus PAH, survivors versus non-survivors as well as before versus after PAH treatment. As for Kruskal-Wallis tests and the following multiple comparisons, FDR was set at Q = 0.05 and 0.1, respectively. For Spearman correlations, FDR was set at Q = 0.1. P values less than the attained FDR-based thresholds were considered statistically significant.

For the regression analyses, p values less than 0.05 were considered statistically significant. Receiver operating characteristic (ROC) curves were plotted to display the diagnostic and prognostic predictive ability of candidate biomarkers. Optimal thresholds and their corresponding sensitivity and specificity were based on Youden’s index. Logistic regression models and COX proportional hazards models were fitted, and their effects were summarized as odds ratios (OR) with 95% confidence intervals (CI) as well as hazard ratios (HRs) with 95% CI, respectively. For the regression analyses, assumptions of at least eight events per variable were used. For the COX-regression models, deaths were regarded as events, whereas having PAH was assumed as an event for the logistic regression models. ROC analyses of the fitted multivariable logistic regression models were based on the probabilities derived from the odds using the following formula: probability = odds/(odds + 1). Delong’s test was used to compare the area under the ROC curves (AUCs) of two ROC-curves.

For internal validation, the optimism adjusted AUCs via a bootstrap procedure (n = 10,000 with resampling) were calculated, based on the model proposed by Harrell et al.^2324^

### Study set-up

The present study focused on identifying diagnostic and prognostic biomarker candidates in PAH. Plasma proteins were stratified according to the presence of a difference between the incident PAH population and the young, healthy control group, qualifying the proteins for further analysis. Such qualified proteins were analysed both in terms of diagnosis/differentiation and prognosis in PAH. For the diagnostic approach, PAH was compared to CTEPH, HFpEF-PH, HFrEF-PH, and a dyspnoea-non-PH control group. Multiple logistic regression models were fitted to combine candidate proteins adjusted for age and sex, followed by a ROC analysis. The prognostic approach involved comparing the levels of survivors versus non-survivors. Proteins displaying a difference between survivors and non-survivors were subsequently analysed with ROC curves to extract the optimal threshold for the Kaplan-Meier analysis. Multivariable COX proportional hazards models were fitted to adjust for age, sex, and the ESC/ERS risk scores, followed by fitting of logistic regression models, on which a ROC curve was based to display the prognostic ability.

## Results

### Population characteristics

Baseline characteristics, some of which have previously been described, are displayed in (Table 1).^25^ Patients with PAH were censored on 8 April 2020. At the end of the follow-up period, 32 out of 48 PAH patients had died. Three patients underwent lung transplantation, whereof two died during follow-up. The median follow-up time was 3.6 (IQR 2.1–4.8) years.

**Table 1.** Patients’ characteristics.

Variable	All PAH patients (n = 48)	PAH before treatment (n = 33)	PAH at early treatment follow-up (n = 33)	CTEPH(n = 20)	HFpEF-PH(n = 31)	HFrEF-PH(n = 36)	Dyspnoea/xHF-non-PH(n = 15)
Female, n (%)	40 (83.3)	29 (87.9)	29 (87.9)	13 (65)	20 (64.5)	7 (19.4)	8 (53.3)
Age (years)	71.5 (64–76)	71 (60.5–76.5)	72 (61–76.5)	75 (70.8–77.8)	76 (69–83)	54 (47.3–59.5)	60 (46–76)
BSA (m^2^)	1.75 (1.59–1.96)	1.73 (1.58–1.79)	1.72 (1.5–1.79)	1.83 (1.75–1.99)	1.89 (1.72–2.13)	2.01 (1.89–2.13)	1.96 (1.65–2.09)
WHO functional class (n) 1/2/3/4	1/9/28/2^h^	1/6/22/2^b^	2/10/15/0^f^	0/6/13/0^a^	–	–	–
Six-minute walking distance (m)	242 (172.5–349)^b^	242 (183.8–345.5)	270 (222.3–337.5)^c^	300 (220–337.5)^c^	–	–	–
NT-proBNP (pg/mL)	2149 (865–3631)^b^	2104 (767–3139)^b^	695 (243–1797)^c^	1473 (199–4538)^b^	1574 (876–2355)^i^	4512 (3613–7230)^f^	1273 (475–2972)^d^
NT-proBNP (AU)	8.8 (4.2–14.3)	7.92 (3.55–11.37)	2.08 (1.25–3)	6.1 (2.01–17.9)	7.56 (4.98–9.94)	30.2 (17.2––43.3)	8.93 (2.42–20.54)
Creatinine (μmol/L)	90 (70.75–113.5)^b^	90 (70–113)^b^	–	88 (73–122.5)^b^	97.5 (78.5–118)^b^	121 (90–145)^a^	93 (80.5–123)^b^
eGFR (mL/min /1.73 m^2^)	58.5 (43.5–68.5)^b^	59.9 (42.1–68.3)^b^	–	56 (46–62.3)^b^	48 (38.3–63)^b^	57 (43–70)^a^	56 (44–77)^b^
*Comorbidities*							
Atrial fibrillation, n (%)	4 (8.3)	2 (6.1)	–	3 (15)	23 (76.7)^a^	14 (38.9)	8 (61.5)^b^
Systemic hypertension, n (%)	17 (35.4)	11 (33.3)	–	11 (55)	20 (74.1)^d^	7 (19.4)	7 (53.8)^b^
Ischemic heart disease, n (%)	7 (14.9)	5 (15.2)	–	1 (5)	6 (25)^g^	6 (16.7)	6 (42.9)^a^
Stroke, n (%)	2 (4.2)	2 (6.1)	–	1 (5)	6 (24)^f^	4 (11.1)	2 (15.4)^b^
Thyroid disease, n (%)	11 (22.9)	10 (30.3)	–	1 (5)	2 (8.3)^g^	3 (8.3)	3 (21.4)^a^
Diabetes mellitus, n (%)	12 (25)	8 (24.2)	–	0 (0)	10 (35.7)^c^	4 (11.1)	3 (21.4)^a^
*Medications*							
Beta-blockers, n (%)	16 (33.3)	9 (27.3)	–	9 (45)	23 (74.2)	35 (97.2)	11 (73.3)
Mineralcorticoid receptor antagonist, n (%)	12 (25)	7 (21.2)	–	3 (15)	8 (25.8)	28 (77.8)	7 (46.7)
Angiotensin-converting enzyme inhibitor, n (%)	10 (20.8)	6 (18.2)	–	2 (10)	11 (35.5)	19 (52.8)	3 (20)
Angiotensin II receptor blocker, n (%)	4 (8.3)	0 (0)	–	2 (10)	10 (32.3)	14 (38.9)	0 (0)
Furosemide, n (%)	11 (22.9)	11 (33.3)	–	7 (35)	6 (19.4)	34 (94.4)	12 (80)
*Haemodynamics*							
mAP (mmHg)	96 (89.4–104)	94.3 (89–103)	86 (80–92)	99 (94–110)	99 (91–106)	79.5 (75.3–88.8)	89 (80–96)
mPAP (mmHg)	43 (37–55)	43 (37–55)	36 (32–48)	42 (35–54)	34 (29–46)	34.5 (29–40.8)	20 (17–22)
mRAP (mmHg)	7 (4–11)	6 (3–9.5)	6 (3–9.5)	5.5 (3.25–8)	10 (7–14)	14.5 (9–17)	6 (2–16)
PAWP (mmHg)	8 (6–11)	6 (5–9.5)	8 (5–11)	9.5 (7–13)	18 (17–23)	25 (19–28)^a^	15 (9–18)
Cardiac output (L)	3.8 (3–5.1)	3.9 (3–5)	4.8 (3.7–6)	4 (3.5–4.7)	4.4 (3.6–5.6)	3.2 (2.8–4)	3.3 (3–4.4)
Cardiac index (L/m^2^)	2.2 (1.8–2.8)	2.2 (1.8–2.8)	2.7 (2.1–3.4)	2.3 (1.9–2.5)	2.3(2.1–2.6)	1.6 (1.4–1.9)	1.9 (1.6–2.3)
Stroke volume index (mL/beat/m^2^)	28.7 (22.6–34.9)	29.2 (23.7–35.4)	37 (29.5–42.8)	30.5 (26.3–32.5)	33.7 (27.8–42.2)	22.5 (18.2–27.2)	29 (25.2–31.9)
LVSWI (mmHg × mL/m^2^)	2484 (2045–3214)	2579 (2054–3188)	2779 (2448–3535)	2508 (2330–3187)	2551 (2150–3209)	1152 (957–1636)^a^	2168 (1650–2716)
RVSWI (mmHg × mL/m^2^)	991 (807–1245)	981 (822–1250)	1258 (917–1396)	1111 (845–1298)	832 (667–1136)	440 (306–649)	382 (195–495)
PVR (WU)	9.5 (6.2–11.8)	9.6 (7.0–12.1)	5.8 (4.3–8.7)	9.3 (5.9–10.8)	3.6 (2.4–5.1)	3 (2.3–3.7)^a^	1.5 (1–2)
PAC (mL/mmHg)	1.065 (0.85–1.49)	1.059 (0.87–1.44)	1.54 (1.19–2.12)	0.96 (0.76–1.34)	1.78 (1.27–2.7)	1.97 (1.68–3.02)	3.33 (2.34–5.34)
TPG (mmHg)	34.5 (25.5–46.5)	37 (29–47)	29 (25–38.5)	36 (27–40.8)	14 (12–23)	10 (7–13)^a^	5 (4–6)
SaO_2_ (%)	91 (87–95)	91 (88–94)	91 (88–95)	89 (86–94)	95 (91–97)	95 (92–96)	96 (95–97)
SvO_2_ (%)	59 (51–66)	62 (54–66)	63 (58–72)	63 (55–68)	63 (57–67)	50 (47–55)	61 (59–69)
a-vO_2_ diff (mL O_2_/L)	53.1 (41.8–63.7)	52.5 (41.5–63.1)	47.1 (35.3–53.9)	54 (49–60.6)	53.1 (46.4–57.8)	77.4 (66.6–86.4)	58.9 (49.2–67.5)

Note: Values are presented as medians (25th–75th percentiles), unless otherwise stated.

AU: arbitrary unit; a-vO_2_ diff: arteriovenous oxygen difference; BSA: body surface area; CTEPH: chronic thromboembolic pulmonary hypertension; eGFR: estimate of glomerular filtration rate; HF: heart failure; HFrEF-PH: pulmonary hypertension due to heart failure with reduced ejection fraction; HFpEF-PH: pulmonary hypertension due to heart failure with preserved ejection fraction; LVSWI, left ventricular stroke work index; mAP: mean arterial pressure; mPAP: mean pulmonary arterial pressure, mRAP, mean right atrial pressure; NT-proBNP: N-terminal pro-brain natriuretic peptide; PAC: pulmonary arterial compliance; PVR: pulmonary vascular resistance; PAH: pulmonary arterial hypertension; RVSWI: right ventricular stroke work index; SaO_2_: arterial oxygen saturation; SvO_2_: mixed venous oxygen saturation; TPG: transpulmonary pressure gradient; WHO: World Health Organization.

^a–i^Indicate n–1 to n–9, respectively.

The incident PAH population comprised patients with idiopathic PAH (IPAH) (n = 21), familial PAH (FPAH) (n = 2), systemic sclerosis-associated PAH (SSc-PAH) (n = 21) and other connective tissue disease-associated PAH (n = 4). The median time to the first, early treatment follow-up was 116 (IQR 90–127) days. Among the 33 PAH patients with treatment follow-up, 23 patients were on monotherapy with either ambrisentan (n = 7), bosentan (n = 5), macitentan (n = 5), sildenafil (n = 4), or tadalafil (n = 2). Nine patients were on combination therapy, including ambrisentan and sildenafil (n = 1), ambrisentan and taladafil (n = 3), bosentan and sildenafil (n = 1), macitentan and sildenafil (n = 2), macitentan and taladafil (n = 1) or macitentan, taladafil and treprostinil (n = 1). One patient was treated with only nifedipine for being an acute nitric oxide vasodilator responder. In addition, some patients were treated with nifedipine for rheumatologic symptoms both at baseline and at treatment follow-up (n = 9), or only at follow-up (n = 1), as previously described.^26^

The median age of the healthy controls was 41 (IQR 27–51) years, and 10 (50%) were female. Upon enrollment, none had a medical history of atrial fibrillation, systemic hypertension, ischemic heart disease, stroke, diabetes mellitus, or thyroid disease (apart from two with a treated thyroid disease).

### Candidate biomarker selection

To identify biomarkers associated with disease and PAH, plasma proteins were compared between the incident PAH patients and the healthy controls. Seventeen plasma proteins out of 42 displayed a difference (p < 0.02; FDR 5%), rendering the basis of further analyses regarding diagnosis/differentiation and prognosis (Table 2).

**Table 2.** Levels of the plasma proteins in the study population.

Plasma protein (AU)	Controls(n = 20)	All PAH patients (n = 48)	PAH before treatment (n = 33)	PAH at early treatment follow-up (n = 33)	CTEPH(n = 20)	HFpEF-HF (n = 31)	HFrEF-PH(n = 33)	Dyspnoea/HF-non-PH(n = 15)
ADAMTS13	28.22 (27.22–29.84)^a^	25.34 (23.69–28.5)	24.25 (23.06–26.82)^d^	24.08 (22.38–26.22)^d^	35.41 (32.74–39.55)	32.48 (29.29–35.38)	33.19 (29.55–37.21)	32.85 (30.37–39.55)
AGRP	7.55 (5.4–8.92)	7.98 (6.41–11.14)	8.41 (6.8–11.49)^d^	9.62 (6.81–11.59)^d^	7.3 (5.83–8.82)	7.08 (5.4–9.08)	8.63 (6.62–11.93)	6.58 (6.14–10.49)
BMP-6	32.6 (26.76–43.23)	39.19 (31.58–48.47)	41.07 (33.04–47.09)^d^	40.01 (32.03–46.56)^d^	36.03 (31.5–42.64)	41.06 (34.25–53.3)	49.6 (37.44–64.43)	41.98 (26.45–57.68)
BMP-9	19.44 (15.25–25.35)	19.4 (14.82–24.39)	21.28 (15.8–25.98)^d^	27.65 (19.58–32.66)^d^	19.07 (14.62–27.46)	19.69 (12.35–25.22)	23.27 (15.81–28.95)	17.72 (15.66–28.1)
CA-VA	2.59 (2.08–6.54)^a^	4.4 (2.87–7.21)	4.8 (2.67–8.24)^d^	4.06 (2.79–6.4)^d^	4.74 (3.45–9.15)	6.19 (4.65–9.48)	7.89 (4.84–12.54)	5.3 (3.81–11.96)
Chymotrypsin-C	1049.81 (740.79–1492.82)	1043.6 (805.8–1221.1)	1040.4 (702.18–1335.2)^d^	1142.69 (747.26–1376.26)^d^	1263.06 (807.05–1577.62)	1193.18 (692.85–1747.52)	1493.91 (887.62–1997.5)	1341.74 (802.56–1951.6)
Dkk-1	285.83 (224.57–461.81)	327.49 (233.65–475.67)	295.98 (235.52–447.73)^d^	319.83 (239.54–489.13)^d^	382.37 (270.87–570.72)	405.04 (272.63–495.06)	274.72 (207.28–364.59)	376.73 (269–538.77)
DLK-1	23.87 (16.62–31.83)	20.26 (13.53–28.74)	21.8 (13.64–28.51)	25.71 (15.99–34.36)	22.6 (16.76–28.76)	27.54 (17.67–39.19)	21.78 (13.58–30.85)	21.65 (19.53–31.47)
DLL1	381.84 (334.45–434.18)^a^^c^	475.97 (381.89–564.79)	460.96 (371.46–573.47)	508.23 (412.82––606.96)^b^^c^	469.73 (403.07–521.8)	575.42 (471.02–657.37)	530.91 (402.59–670.99)^c^	489.02 (409.87–597.73)
Follistatin	1863.81 (1475.33–2447.34)^a^	2719.9 (1879–3600.5)	2459.9 (1846.8–3188.9)^d^	2457.15 (2125.61–3274.91)^d^	3176.55 (2462.52–4377.02)	3544.08 (2754.97–4446.05)	3576.51 (2631.02–4798.58)	3062.01 (2555.74–4020.52)
Gastric intrinsic factor	41.43 (29.04–57.04)	50.09 (32.41–87.01)	49.15 (29.68–90.17)^d^	63.37 (29.66–91.76)^d^	54.73 (44.35–96.65)	64.93 (36.43–133.92)	42.32 (28.08–71.07)	83.86 (65.53–156.91)
HAOX1	11.41 (6.27–20.12)^a^	21.77 (12.01–44.41)	19.86 (8.19–43.98)^d^	15.51 (8.26–27.95)^d^	20.03 (12.56–34.45)	18.35 (10.74–52.98)	47.12 (19.56–88.04)	18.17 (12.93–26.79)
Heme oxygenase 1	1295.78 (1049.27–1665.08)	1524.6 (1201.8–1937.3)	1369.2 (1133.8–1830.4)^d^	1413.02 (1169.62–1681.88)^d^	1892.56 (1548.85–2315.1)	1783.02 (1311.44–2251.95)	1752.96 (1472.15–2149.07)	1560.54 (1325.06–2200.47)
HspB1	1535.16 (1443.79–1692.38)	1458.4 (1347.8–1541.5)	1404.2 (1283.3–1510.2)^d^	1401.46 (1271.09–1466.65)^d^	1529.83 (1424.71–1631.03)	1545.24 (1406.76–1691.22)	1473.21 (1348.6–1629.08)	1504.82 (1441.09–1703.62)
IDUA	23.79 (20.26–32.83)	25.27 (21.56–28.6)	24.79 (21.5–27.94)^d^	24.54 (18.7–30.52)^d^	32.44 (26.35–35.49)	33.1 (25.59–40.98)	30 (23.15–35.02)	31.77 (26.78–39.26)
MAD homolog 5	8.06 (6.89–8.6)^c^	8.24 (7.71–8.69)	8.2 (7.63–8.77)	8.52 (7.92–8.71)^c^	9.35 (8.89–10.01)	9.26 (8.75–9.91)	9.24 (8.27–10.1)	9 (7.71–9.29)
MST-1	28.74 (14.29–35.72)	20.39 (12.87–33.81)	18.2 (11.21–31.29)^d^	19.78 (9.11–27.75)^d^	20.49 (15.08–26.14)	26.47 (20.09–40.46)	16.63 (9.62–35.15)	31.35 (18.41–38.68)
NEMO	131.54 (66.03–268.4)	123.97 (63.22–214.71)	91.89 (60.78–177.1)^d^	87.21 (43.08–167.46)^d^	68.53 (50.03–87)	125.89 (67.08–210.01)	82.67 (40.45–135.77)	105.45 (57.46–231.79)
Notch 3	7.95 (7.17–9.36)^a^	10.87 (9.05–16.39)	12.16 (7.81–15.09)	10.94 (8.04–13.91)	13.18 (10.33–17.46)	13.79 (10.55–18.42)	15.06 (11.46–19.94)	10.82 (9.04–14.55)
NT-proBNP	1.13 (1.12–1.17)^a^	8.79 (4.21–14.25)	7.67 (3.67–10.65)	4.22 (2.38–8.07)	6.05 (2.01–17.89)	7.56 (4.98–9.94)	30.2 (17.2–43.32)	8.93 (2.42–20.54)
PAI-1	43.94 (29.07–58.82)	49.48 (29.95–89.03)	42.08 (25.75–81.82)	41.13 (27.3–79.05)	89.71 (58.88–170.21)	68.96 (39.76–118.56)	58.66 (43.84–102.31)	89.77 (42.44–116.42)
PAR-1	170.69 (127.59–227.53)	179.8 (133.9–223.78)	160.44 (137.28–183.76)^d^	176.45 (143.64–205.79)^d^	195.47 (163.06–246.63)	221.4 (198.44–268.02)	201.8 (153.7–252.93)	214.59 (182.02–279.25)
PARP-1	4.44 (3.61–6.53)	4.87 (4.32–6.76)	5.58 (4.76–7.56)^d^	5.36 (3.97–7.09)^d^	4.47 (3.65–5.44)	3.82 (3.28–5.05)	4.55 (3.65–6.16)	4.61 (3.43–5.51)
PD-L2	4.55 (3.96–5.35)^a^	5.89 (5.3–7.39)	5.8 (5.4–7.41)^d^	6.44 (5.07–8.1)^d^	6.04 (4.96–6.73)	6.79 (5.7–7.62)	6.89 (5.81–8.47)	6.42 (5.84–8.33)
PPY	21.98 (10.5–57.75)	37.31 (26.07–74.65)	36.98 (18.83–64.89)	34 (16.87–72.56)^c^	41.78 (26.63–103.93)	57.16 (27.18–128.61)	82.65 (30.74–133.03)^c^	39.29 (10.99–90.56)
PRSS27	198.47 (129.08–246.06)	193.21 (150.93–251.12)	169.46 (145.39–251.74)^d^	194.06 (169.38–259.22)^d^	197.4 (161.91–242.14)	221.7 (165.83–268.35)	179.09 (152.72–220.17)	231.42 (166.52–315.6)
R-spondin-3	7.66 (6.58–9.06)^a^^c^	13.11 (10.67–17.74)	13.72 (10.38–18.74)	14.41 (11.59–19.69)^c^	16.23 (11.33–20.28)	15.36 (12.67–24.72)	17.25 (10.78–18.64)^c^	12.2 (9.7–14.74)
SEZ6L	25.08 (20.84–27.74)^c^	25.45 (21.09–30.05)	24.6 (20.79–30.35)	24.73 (22.07–30.23)^d^	28.53 (25.29–30.57)	26.21 (21.32–30.25)	27.38 (22.65–33.07)^c^	26.77 (25.06–32.13)
SOD2	325.29 (271.32–381.7)	318.29 (284.53–366.37)	319.17 (287.29–359.58)^d^	314.7 (269.42–357.49)^d^	301.81 (265.47–325.98)	318.54 (294.37–365.48)	357.77 (320.79–386.34)	324.38 (310.57–378.26)
Somatotropin	184.58 (85.58–332.1)^a^	647.95 (208.05–1619.5)	499.17 (200.67–1542.5)^d^	391 (207.15–1259.06)^d^	288.43 (134.54–1547.44)	398.39 (204.73–2211.41)	1242.33 (797.19–2993.53)	1111.7 (265.57–1600.59)
Spondin-1	2.92 (2.72–3.24)^a^	3.99 (3.29–4.45)	4.02 (3.21–4.4)	3.9 (3.16–4.34)	3.94 (3.66–5.06)	4.86 (4.06–5.76)	4.87 (3.84–6.08)	3.99 (3.73–6)
Spondin-2	292.63 (273.99–323.76)^a^	406.59 (384.93–423.5)	406.85 (387.29–420.88)^d^	408.93 (369.37–430.49)^d^	435.07 (404.5–483.84)	450.63 (394.46–496.62)	454.42 (421.62–512.82)	423.48 (403.36–455.83)
TFPI	265.49 (213.07–306.6)	215.26 (167.45–262.73)	217.18 (141.02–311.19)	234.44 (121.11–289.79)	217.54 (192.76–251.52)	245.13 (206.62–297.79)	252.52 (222.26–298.97)	239.14 (218.03–287.02)
TFPI-2	110.53 (101.24–125.36)^a^^c^	245.33 (184.2–302.19)	255.02 (180.97–292.33)	220.04 (171.18–284.71)^c^	204.08 (179.7–280.67)	209.89 (177.51–255.81)	244.82 (208.73–383.07)^c^	212.24 (182.32–258.45)
TGFBRII	82.69 (73.05–98.87)^a^^c^	109.94 (80.58–151.95)	102.38 (83.27–149.75)	127.5 (94.57–154.22)^b^^c^	110.41 (94.74–120.52)	139.95 (122.09–195.97)	106.44 (85.36–156.95)^c^	117.85 (107.25–134.93)
Thrombopoietin	4.56 (3.86–6.22)	4.54 (3.72–5.58)	4.38 (3.66–5.44)^d^	4.71 (3.49–5.68)^d^	6.02 (4.82–6.66)	5.76 (4.77–6.94)	4.82 (4.12–5.75)	5.67 (4.41–7.44)
Thrombomodulin	305.4 (268.36–366.27)	265.54 (220.96–352.78)	270.4 (217.38–333.52)^d^	309.78 (254.05–379.61)^d^	323.8 (263.61–383.08)	417.62 (323.56–524.98)	340.12 (249.09–505.86)	341.85 (306.66–399.67)
Tissue factor	31.95 (29.48–37.68)^a^	41.83 (31.23–49.51)	40.36 (30.81–51.29)^d^	41.08 (30.33–56)^d^	60.21 (49.04–70.87)	58.38 (48.42–69.06)	53.25 (41.47–64.86)	49.52 (43.14–73.09)
t-PA	27.65 (21.98–39.48)^a^	51.01 (39.16–71.21)	53.52 (31.23–71.68)	42.42 (28.48–59.58)	61.05 (40.09–80.3)	46.84 (37.03–76.91)	46.94 (39.64–67.92)	42.06 (35.52–66.17)
u-PA	20.18 (18.95–22.46)	19.06 (14.95–26.11)	20.82 (14.88–26.28)	19.63 (15.62–24.07)	18.63 (16.6–21.14)	20.21 (16.46–24.54)	21.5 (17.15–29.07)	20.64 (17.26–23.61)
uPAR	10.81 (9.87–11.97)^a^	19.3 (16.86–22.4)	19.33 (15.93–22.17)	18.74 (14.02–25.33)	21.92 (16.29–24.66)	20.66 (17.42–26.45)	19.1 (15.8–30.56)	17.28 (14.01–23.25)
vWF	65.84 (46.45–90.6)^a^	147.22 (93.61–224.16)	134.97 (73.22–249.08)	101.85 (67.39–261.46)	135.73 (111.36–210.46)	142.29 (97.66–214.15)	113.63 (88.49–188.45)	150.97 (96.09–204.86)
WIF-1	40.36 (33–45.71)^c^	43.77 (30.29–57.93)	43.92 (32.48–58.05)	46.81 (33.8–52.64)^c^	40.75 (34.87–50.64)	39.8 (34.79–60.05)	59.02 (50.17–75.1)^d^	43.52 (37.49–60.28)

ADAMTS13: a disintegrin and metalloproteinase with thrombospondin motifs 13; AGRP: agouti-related protein; AU: arbitrary unit; BMP: bone morphogenetic protein; CA-VA: carbonic anhydrase VA; CTEPH: chronic thromboembolic pulmonary hypertension; Dkk-1: dickkopf-related protein 1; DLK-1: protein delta homolog 1; DLL1: delta-like protein 1; HAOX1: hydroxyacid oxidase 1; HF: heart failure; HFrEF-PH: pulmonary hypertension due to heart failure with reduced ejection fraction; HFpEF-PH: pulmonary hypertension due to heart failure with preserved ejection fraction; HspB1: heat shock protein beta-1; IDUA: alpha-L-iduronidase; Mad homolog 5: mothers against decapentaplegic homolog 5; MST-1, serine/threonine-protein kinase 4; NEMO: NF-kappa-B essential modulator; Notch 3: neurogenic locus notch homolog protein 3; NT-proBNP: N.-terminal pro-brain natriuretic peptide; PAH: pulmonary arterial hypertension; PAI-1: plasminogen activator inhibitor 1; PAR-1: proteinase-activated receptor 1; PARP-1: poly [ADP-ribose] polymerase 1; PD-L2: programmed cell death 1 ligand 2; PPY: pancreatic prohormone; PRSS27: serine protease 27; SEZ6L: seizure 6-like protein; SOD2; superoxide dismutase [Mn] mitochondrial; TFPI: tissue factor pathway inhibitor; (TGFBRII): TGF-beta receptor type-2; t-PA, tissue-type plasminogen activator; u-PA: urokinase-type plasminogen activator; uPAR: urokinase plasminogen activator surface receptor; vWF: von Willebrand factor; WIF-1:, Wnt inhibitory factor 1.

^a^Indicates significant difference in PAH versus controls (p < 0.026; false discovery rate 5%).

^b^Indicates significant difference after versus before treatment (p < 0.001; false discovery rate 5%).

^c^Indicates n = 1.

^d^Indicates n = 2.

### Plasma ADAMTS13, tissue factor, and spondin-2 differentiates patients with PAH

**Figure 1.**
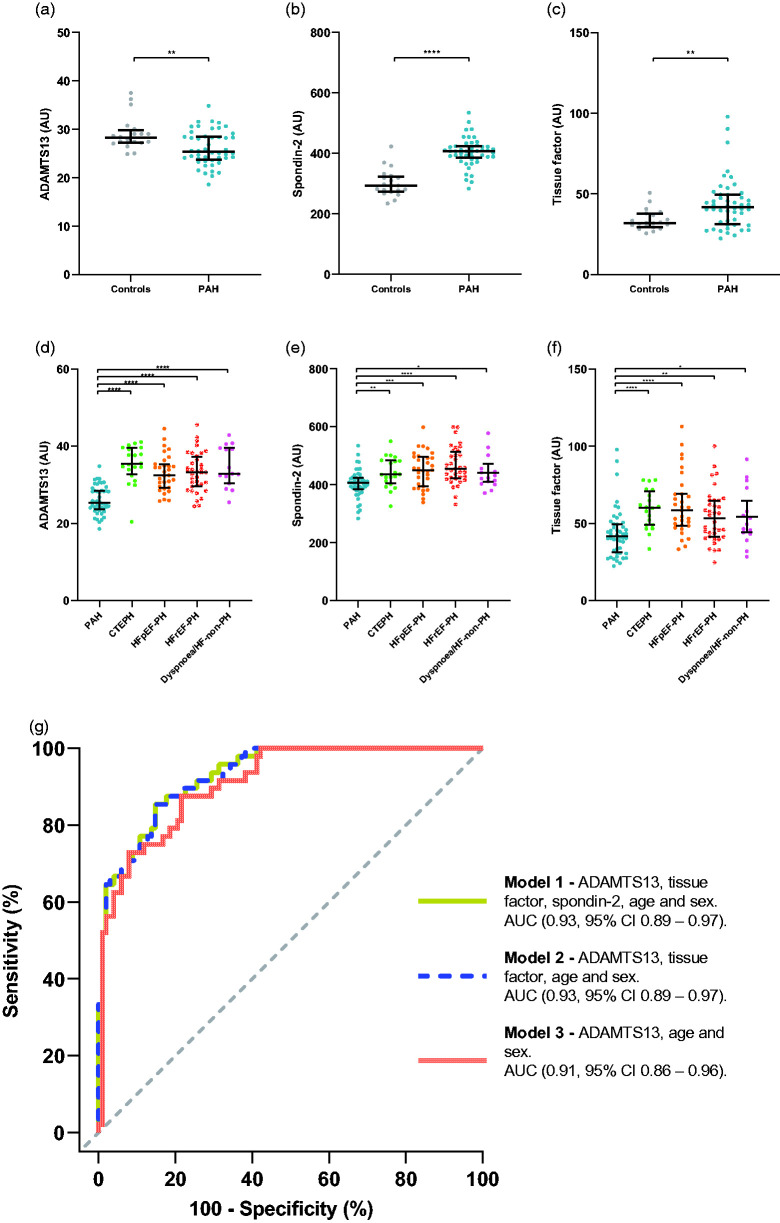
Biomarker candidates for differentiation and diagnosis of pulmonary arterial hypertension. In (a–c), levels of PAH patients versus controls are depicted in scatterplots for ADAMTS13, spondin-2 and tissue factor. D-F, levels of ADAMTS13, spondin-2 and tissue factor in PAH, compared to CTEPH, HFpEF-PH, HFrEF-PH, and Dyspnoea/HF-non-PH. G, ROC-curves (based on multivariable logistic regression models) displaying the discriminatory ability of each model in identifying PAH from the other disease groups. (a–c), statistical significance was considered p < 0.015; false discovery rate (FDR<0.05) and in (d–f), p < 0.05;(FDR<0.1). *Indicates p < 0.05, **p < 0.01, ***p < 0.001, ****p < 0.0001. ADAMTS13: a disintegrin and metalloproteinase with thrombospondin motifs 13; AU: arbitrary unit; CTEPH: chronic thromboembolic pulmonary hypertension; HF: heart failure; HFrEF-PH: pulmonary hypertension due to heart failure with reduced ejection fraction; HFpEF-PH: pulmonary hypertension due to heart failure with preserved ejection fraction; PAH: pulmonary arterial hypertension; PH: pulmonary hypertension.

Out of the 17 qualified plasma proteins, plasma ADAMTS13, tissue factor, and spondin-2-differentiated PAH from the other disease groups (Figure 1). Specifically, plasma ADAMTS13 levels were lower in PAH patients compared to CTEPH (p < 0.0001), HFpEF-PH (p < 0.0001), HFrEF-PH (p < 0.0001), Dyspnoea/HF-non-PH (p < 0.0001) as well as healthy controls (p = 0.0055) (Figure 1(a) and (d)). Plasma tissue factor and spondin-2 displayed a similar pattern, with the PAH group having the lowest levels compared to the other disease groups (Figure 1(e) and (f)). However, the levels of both tissue factor and spondin-2 in PAH were intermediate between the healthy controls and the disease groups (Figure 1(b) and (c)), (Table 2). Additionally, no difference was observed in either ADAMTS13 (p = 0.22), tissue factor (p = 0.22) or spondin-2 (p = 0.44) before versus after treatment initiation in patients with PAH (Table 2).

For assessment of PAH discrimination, ROC analyses were performed on plasma ADAMTS13, tissue factor and spondin-2, in which PAH was compared to all other disease groups. ADAMTS13 displayed the highest unadjusted AUC (0.90, 95% CI 0.85–0.95) (Table 3), followed by tissue factor (0.75, 95% CI 0.66–0.84) and spondin-2 (0.747, 95% CI 0.67–0.83). Next, three logistic regression models were fitted with subsequent ROC analyses to assess the discriminatory ability of all three candidate biomarkers, age, and sex (model 1); ADAMTS13, tissue factor, age and sex (model 2), and ADAMTS13, age and sex (model 3) (Table 3). Model 1 displayed the highest mean AUC (0.93, 95% CI 0.89–0.97) and model 3 the lowest (0.91, 95% CI 0.86–0.96) (Figure 1(g)). However, model 1 was not superior to model 3 in terms of AUC (p = 0.095). Using the odds derived cut-off (≥0.269), model 3 exhibited a sensitivity of 87.5% and a specificity of 78.4% for identifying PAH among the other disease groups. Internal validation of model 3 yielded an optimism-adjusted mean AUC of 0.90 (Table 3). In a subgroup analysis of IPAH and SSc-PAH, we found no difference in ADAMTS13 levels (p = 0.29). In addition, ADAMTS13 correlated with PAWP (r_s_ = 0.33, p = 0.021) (Table 4).

**Table 3.** Candidate biomarkers and related models for diagnosis and differentiation of pulmonary arterial hypertension.

ROC analyses of diagnosis and differentiation plasma protein candidates in PAH			Unadjusted AUC (95 % CI)		Cut-off^a^ (AU)	Sensitivity (%)	Specificity (%)
ADAMTS13 (AU)			0.90 (0.85–0.95)		<28.56	77.08	86.27
Tissue factor (AU)			0.75 (0.66–0.84)		<45.9	68.75	73.53
Spondin-2 (AU)			0.75 (0.67–0.83)		<424.09	77.08	67.65
Multiple logistic regression models and roc analyses	OR (95% CI)	p	AUC (95% CI)	Optimism-adjusted AUC^b^	Cut-off^a^ (probability)	Sensitivity (%)	Specificity (%)
Model 1
ADAMTS13 (AU)	0.68 (0.56–0.78)	<0.0001	0.93 (0.89–0.97)	0.916	≥0.307	85.42	85.29
Tissue factor (AU)	0.95 (0.91–0.99)	0.024					
Spondin-2 (AU)	1.00 (0.99–1.01)	0.71					
Age (y)	1.01 (0.97–1.05)	0.66					
Sex (F)	4.40 (1.49–14.3)	0.0095					
Model 2
ADAMTS13 (AU)	0.67 (0.56–0.77)	<0.0001	0.93 (0.89–0.97)	0.918	≥0.313	85.42	85.29
Tissue factor (AU)	0.95 (0.91–0.98)	0.0021					
Age (y)	1.01 (0.97–1.05)	0.67					
Sex (F)	4.51 (1.54–14.58)	0.0079					
Model 3
ADAMTS13 (AU)	0.67 (0.58–0.76)	<0.0001	0.91 (0.86–0.96)	0.903	≥ 0.269	87.5	78.43
Age (y)	0.99 (0.95–1.02)	0.45					
Sex (F)	4.94 (1.77–15.18)	0.0033					
Model 3 versus model 2	–	0.095	–	–	–	–	–

ADAMTS13: a disintegrin and metalloproteinase with thrombospondin motifs 13; AU: arbitrary unit; AUC: area under the ROC curve; CI: confidence interval; PAH: pulmonary arterial hypertension.

^a^Indicates that the optimal cut-off was calculated using Youden’s index.

^b^Indicates the optimism-adjusted AUC obtained from internal validation using bootstrap (n = 10,000 with resampling).

**Table 4.** Correlations of ADAMTS13 and vWF with haemodynamics and demographic parameters.

Variable	ADAMTS13 (AU)	vWF (AU)	DLL1 (AU)	TGFBRII (AU)
	r_s_ (p)	r_s_ (p)	r_s_ (p)	r_s_ (p)
World Health Organization functional class	–0.0053 (0.97)^a^	0.26 (0.11)^a^	–0.071 (0.67)^a^	–0.072 (0.66)^a^
Six-minute walking distance (m)	0.058 (0.70)^b^	–0.11 (0.46)^b^	–0.27 (0.071)^b^	–0.17 (0.25)^b^
NT-proBNP (AU)	0.17 (0.25)	0.23 (0.12)	0.18 (0.22)	0.11 (0.44)
SvO_2_ (%)	–0.15 (0.31)	–0.014 (0.93)	0.058 (0.7)	0.11 (0.45)
Mean Pulmonary arterial pressure (mmHg)	0.18 (0.22)	0.031 (0.83)	–0.31 (0.031)	–0.39 (0.0056)^c^
Mean right atrial pressure (mmHg)	0.22 (0.13)	0.054 (0.72)	0.18 (0.22)	0.13 (0.36)
Pulmonary arterial wedge pressure (mmHg)	0.33 (0.021)	–0.012 (0.94)	0.26 (0.075)	0.21 (0.14)
Cardiac index (L/m^2^)	–0.098 (0.51)	–0.023 (0.88)	0.17 (0.25)	0.22 (0.14)
Pulmonary arterial compliance (mL/mmHg)	–0.11 (0.46)	0.13 (0.39)	0.25 (0.089)	0.35 (0.013)
Pulmonary vascular resistance (WU)	0.12 (0.40)	–0.046 (0.76)	–0.39 (0.0059)^c^	–0.5 (0.00025)^c^
Left ventricular stroke work index (mmHg × mL/m^2^)	–0.021 (0.89)	–0.13 (0.40)	0.029 (0.85)	0.11 (0.46)
Right ventricular stroke work index (mmHg × mL/m^2^)	–0.029 (0.85)	–0.048 (0.75)	–0.16 (0.27)	–0.19 (0.18)
eGFR (mL/min/1.73 m^2^)	0.17 (0.26)^b^	–0.092 (0.54)^b^	–0.61 (<0.0001)^b^^c^	–0.52 (0.00019)^b^^c^

ADAMTS13: a disintegrin and metalloproteinase with thrombospondin motifs 13; AU: arbitrary unit; DLL1: delta-like protein 1; eGFR: creatinine-based estimate of glomerular filtration rate; NT-proBNP: N-terminal pro-brain natriuretic peptide; r_s_: Spearman’s rank correlation coefficient; SvO_2_: mixed venous oxygen saturation; TGFBRII: TGF-beta receptor type-2; WU: wood units; vWF: von Willebrand factor.

^a^Indicates n = 40 pairs.

^b^Indicates n = 46 pairs.

^c^Indicates statistical significance (p < 0.006; false discovery rate 10%).

### Plasma von Willebrand factor predicts prognosis in patients with PAH

**Figure 2.**
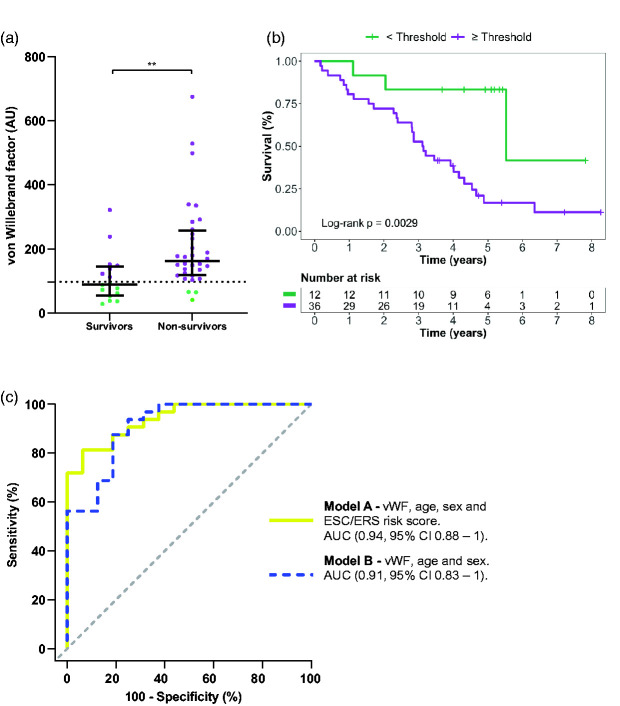
Plasma von Willebrand factor in relation to survival and prognosis prediction in pulmonary arterial hypertension. (a) Scatterplots of von Willebrand factor levels of incident PAH patients, comparing survivors vs. non-survivors. The optimal threshold using Youden’s index for discrimination is plotted as a dotted line. (b) using the optimal threshold, survival of patients was plotted using the Kaplan-Meier method. (c) ROC-curves (based on multivariable logistic regression models), displaying the discriminatory ability of each model in prognostication of PAH patients, based on the follow-up time, median (IQR): 3.6 (2.1–4.8) years. AU: arbitrary unit; PAH: pulmonary arterial hypertension; vWF: von Willebrand factor.

The incident PAH population was divided into survivors and non-survivors and compared for disparities in plasma expression (Table 5). Out of the 17 plasma proteins, only four displayed a difference between survivors and non-survivors, i.e. vWF (p = 0.0016), spondin-1 (p = 0.0056), uPAR (p = 0.006), and Notch 3 (p = 0.011). Univariable COX-regression analysis of these proteins as well as age, sex, and ESC/ERS risk score demonstrated that the only significant predictors were vWF (HR 1.002, 95% CI 1–1.005; p = 0.045) and age (HR 1.055, 96% CI 1.015–1.097). For vWF, a ROC analysis was performed to find the best discriminatory cut-off (96.91 AU) for the Kaplan-Meier analysis, where patients with PAH above or equal to the threshold displayed worse survival compared to patients with PAH below the threshold (p = 0.0029) (Figure 2(a) and (b)). In a multivariable COX-regression analysis, vWF remained significant (p = 0.041) after adjusting for age, sex, and ESC/ERS risk score (Table 6).

**Table 5.** Plasma proteins’ levels of survivors versus non-survivors of patients with PAH.

Plasma protein (AU)	Survivors (n=16)	Non-survivors (n=32)	p
ADAMTS13	24.98 (24.25–30)	25.55 (23.28–28.29)	0.43
CA-VA	5.08 (3.37–7.18)	4.1 (2.77–7.76)	0.33
DLL1	458.15 (350.89–570.22)	484.1 (414.88–564.8)	0.44
Follistatin	2965 (2034–3883)	2644.06 (1847.19–3384.26)	0.43
HAOX1	24.39 (14.65–74.79)	18.81 (8.56–38.53)	0.14
Notch 3	9.52 (6.25–10.72)	12.2 (9.9–18.48)	0.01117^a^
NT-proBNP	7.29 (2.37–16.93)	9.57 (4.66–13.64)	–
PD-L2	5.73 (5.06–7.2)	5.89 (5.32–7.6)	0.70
R-spondin-3	12.61 (8.94–14.9)	13.33 (10.95–21.39)	0.095
Somatotropin	1159 (268–2308)	533.23 (203.14–1284.61)	0.082
Spondin-1	3.11 (2.51–4.18)	4.13 (3.69–4.86)	0.0056^a^
Spondin-2	403.25 (385.34–423.45)	406.59 (381.87–425.01)	0.94
TFPI-2	242.15 (182.64–338.83)	255.09 (184.2–297.47)	0.96
TGFBRII	107.35 (67.59–148.83)	109.94 (84.97–152.28)	0.60
Tissue factor	39.25 (28.95–45)	43.91 (34.7–50.34)	0.24
t-PA	42.91 (24.78–62.42)	57.1 (41.73–72.14)	0.064
uPAR	16.94 (11.98–20.06)	20.4 (17.84–25.18)	0.0060^a^
vWF	88.74 (54.76–145.1)	161.41 (118.6–256.84)	0.0016^a^

ADAMTS13: a disintegrin and metalloproteinase with thrombospondin motifs 13; AU: arbitrary unit; CA-VA: carbonic anhydrase VA; DLL1: delta-like protein 1; HAOX1: hydroxyacid oxidase 1; Notch 3: neurogenic locus notch homolog protein 3; NT-proBNP: N-terminal pro-brain natriuretic peptide; PD-L2: programmed cell death 1 ligand 2; TFPI: tissue factor pathway inhibitor; (TGFBRII): TGF-beta receptor type-2; t-PA: tissue-type plasminogen activator; uPAR: urokinase plasminogen activator surface receptor; vWF: von Willebrand factor.

^a^Indicates statistically significant comparisons (p <0.012; false discovery rate 5%).

**Table 6.** Candidate biomarkers and related models for prediction of pulmonary arterial hypertension prognosis.

ROC analyses of prognostic plasma protein candidates in PAH			P
von Willebrand factor	AUC (95 % CI)	0.78 (0.63–0.92)	
	Cut-off^a^ (AU)	≥96.91	
	Sensitivity, specificity (%)	90.63, 56.25	
Spondin-1	AUC (95 % CI)	0.74 (0.57–0.92)	
	Cut-off^a^ (AU)	≥3.32	
	Sensitivity, specificity (%)	93.75, 62.5	
uPAR	AUC (95 % CI)	0.74 (0.58–0.90)	
	Cut-off^a^ (AU)	≥17.13	
	Sensitivity, specificity (%)	87.5, 56.25	
Notch 3	AUC (95 % CI)	0.72 (0.57–0.88)	
	Cut-off^a^ (AU)	≥10.76	
	Sensitivity, specificity (%)	81.25, 68.75	
**Univariable cox regression**		HR (95% CI)	
	von Willebrand factor (AU)	1.002 (1–1.005)	0.045
	Spondin-1 (AU)	1.15 (0.94–1.41)	0.18
	uPAR (AU)	1.029 (0.99–1.068)	0.14
	Notch 3 (AU)	1.045 (0.98–1.11)	0.16
	Age (y)	1.055 (1.015–1.097)	0.0068
	Sex (F)	0.34 (0.14–0.81)	0.015
	ESC/ERS risk score	1.38 (0.69–2.77)	0.36
**Multivariable cox regression**		HR (95 % CI)	
Model 1	von Willebrand factor (AU)	1.002 (1–1.004)	0.041
	Age (y)	1.073 (1.027–1.12)	0.0018
	Sex (F)	0.21 (0.078–0.54)	0.0014
	ESC/ERS risk score exact	0.83 (0.37–1.85)	0.64
Model 2	Spondin-1 (AU)	1.17 (0.94–1.45)	0.17
	Age (y)	1.069 (1.023–1.12)	0.0029
	Sex (F)	0.16 (0.061–0.44)	0.00034
	ESC/ERS risk score	0.92 (0.44–1.93)	0.82
Model 3	uPAR (AU)	1.026 (0.98–1.075)	0.28
	Age (y)	1.067 (1.022–1.12)	0.0034
	Sex (F)	0.17 (0.062–0.45)	0.00046
	ESC/ERS risk score	0.79 (0.34–1.85)	0.59
Model 4	Notch 3 (AU)	1.032 (0.97–1.097)	0.31
	Age (y)	1.067 (1.022–1.12)	0.0032
	Sex (F)	0.18 (0.07–0.48)	0.00058
	ESC/ERS risk score	0.93 (0.44––1.98)	0.86
**Multiple logistic regression models and ROC analyses**			
Model A	Independent variables		
	von Willebrand factor (AU), OR (95% CI)	1.045 (1.018–1.091)	0.0097
	Age (y), OR (95% CI)	1.47 (1.15–2.18)	0.014
	Sex (F), OR (95% CI)	4.02 x 10^–6^ (8.67 × 10^–12^–0.02)	0.02
	ESC/ERS risk score	0.038 (0.00063–0.65)	0.057
	AUC (95 % CI)	0.94 (0.88–1)	
	Cut-off^a^ (probability)	≥0.717	
	Sensitivity, specificity (%)	81.25, 93.75	
	Optimism adjusted AUC^b^	0.896	
Model B	Independent variables		
	von Willebrand factor (AU), OR (95% CI)	1,024 (1.01–1.044)	0.005
	Age (y), OR (95% CI)	1.2 (1.075–1.42)	0.011
	Sex (F), OR (95% CI)	0.0051 (1.47 x 10^–5^–0.31)	0.037
	AUC (95 % CI)	0.91 (0.83–1)	
	Cut-off^a^ (probability)	≥0.619	
	Sensitivity, specificity (%)	87.5, 81.25	
	Optimism adjusted AUC^b^	0.877	
Model A versus model B		–	0.3

AU: arbitrary unit; AUC: area under the ROC curve; CI: confidence interval; ESC: European Society of Cardiology; ERS: European Society of Cardiology and the European Respiratory Society; Notch 3: neurogenic locus notch homolog protein 3; PAH: pulmonary arterial hypertension; uPAR: urokinase plasminogen activator surface receptor.

^a^Indicates that the optimal cut-off was calculated using Youden’s index.

^b^Indicates the optimism-adjusted AUC obtained from internal validation using bootstrap (n=10,000 with resampling).

Next, to assess the prognostic ability of baseline vWF levels in patients with PAH and the additional value of the ESC/ERS risk score, two logistic regression models were fitted, including model A (vWF, age, sex, and ESC/ERS risk score) and model B (vWF, age, and sex). For the median follow-up of 3.6 years, model A displayed an AUC of (0.94, 95% CI 0.88–1), whereas model B had an AUC of (0.91, 95% CI 0.83–1) (Figure 2(c)). There was no difference between model B and A (p = 0.3). Internal validation displayed a mean AUC of 0.90 and 0.88 for model A and B, respectively (Table 6). Moreover, no difference was observed in vWF (p = 0.66) before treatment versus after treatment initiation in patients with PAH (Table 2). Furthermore, vWF did not correlate with haemodynamics or other demographic characteristics as shown in (Table 4).

## Discussion

Despite the advances in PAH treatment over the past decades and an established link between diagnostic delay and increased mortality, the median time from symptom onset to diagnosis has not improved.^910^ Early diagnosis of PAH remains challenging, due to non-specific symptoms such as dyspnoea and lethargy.^9^ Beyond increasing the public awareness and education of primary care providers to improve access to initial testing of patients with dyspnoea and/or PAH,^9–11^ adjuvant biomarker screening of certain at-risk individuals for earlier initial testing and referral to expert PH centers may be crucial to minimize the diagnostic delay and improve outcome. In the present study, we have identified plasma ADAMTS13 as a discriminator of incident PAH patients from other dyspnoea-associated disease groups including CTEPH, HFpEF-PH, HFrEF-PH, and HF without PH. Furthermore, plasma levels of ADAMTS13, after adjusting for age and sex, displayed a sensitivity of 87.5% and a specificity of 78.4% for identifying PAH among the other disease groups.

**Figure 3.**
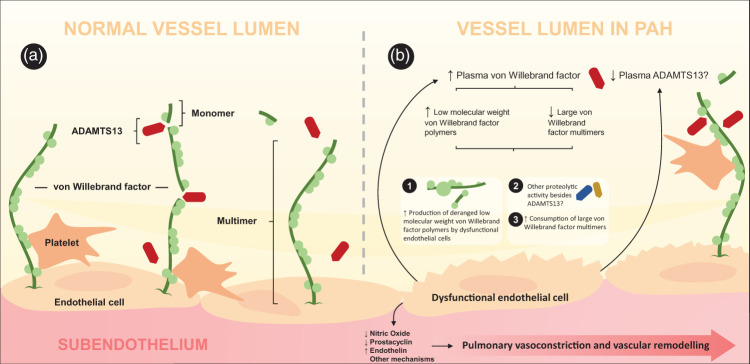
von Willebrand factor and ADAMTS13 in pulmonary arterial hypertension. Decreased availability of nitric oxide and prostacyclin as well as increased endothelin establishes the hallmarks of endothelial dysfunction in PAH, with impact on pulmonary vasoconstriction and vascular remodelling. Hypothetically, dysfunctional endothelium may furthermore be an important cause of higher plasma von Willebrand factor and lower plasma levels of ADAMTS13. Plasma ADAMTS13 cleaves large von Willebrand factor (vWF) multimers into light weight polymers, rendering reduced anticoagulant activity. The elevated levels of plasma vWF consist of paradoxically low levels of large multimers and high levels of low molecular weight polymers, despite low levels of plasma ADAMTS13. This may be due to increased consumption of large vWF multimers, increased release of deranged vWF polymers characterized by low biological activity, or potentially by other proteolytic activity besides that of ADAMTS13.

ADAMTS13 is a circulating metalloproteinase which cleaves ultra large vWF, thereby maintaining the balance between haemostasis and thrombosis.^27^ vWF plays a crucial role in primary haemostasis by mediating platelet adhesion to the site of vascular injury.^2728^ Cleavage of ultra large vWF multimers by ADAMTS13 renders small vWF fragments with less procoagulant activity.^19^ An inability to cleave ultra large vWF, due to deficiency of ADAMTS13, leads to thrombotic thrombocytopenic purpura, a disorder in which occlusion of arterioles and capillaries occur due to accumulation of ultra large vWF and excessive platelet aggregation.^27^ Furthermore, disproportionate levels of low circulating ADAMTS13 and high plasma vWF are risk factors associated with development of cardiovascular and inflammatory disorders including myocardial infarction,^27^ preeclampsia,^29^ and ischemic stroke.^30^ ADAMTS13 is produced by hepatic stellate cells and vascular endothelial cells, where the latter may significantly contribute to plasma levels of ADAMTS13, considering the massive surface they cover. Thus, we hypothesize that the reduced levels of ADAMTS13 in PAH may be related to the dysfunctional endothelium in PAH (Figure 3).

A recent study proposed a dysregulated ADAMTS13-vWF axis in patients with CTEPH and assessed plasma ADAMTS13 levels in IPAH. The authors found no difference in ADAMTS13 between IPAH patients and healthy controls,^31^ inconsistent with our results. Given that our PAH population encompasses different PAH aetiologies, we performed a subgroup analysis of IPAH and SSc-PAH and found no difference in ADAMTS13 levels (p = 0.29). The discrepancy in controls’ levels versus IPAH patients with regard to ADAMTS13, could thus be ascribed to the different sampling time in relation to the PAH diagnosis, as we sampled during the diagnostic RHC and their sampling delay from the diagnosis of IPAH was not reported quantitively.^31^ Moreover, the inconsistency could also be attributed to a putative effect of treatment response. It is possible that the absence of a disparity in plasma ADAMTS13 before versus after treatment in the present study could be due to the short time interval of follow-up. Although it remains speculative, further, larger studies would be of value to validate our results and confirm the consistency of ADAMTS13 in PH. In light of our findings, however, ADAMTS13 may be a promising future biomarker for early identification of PAH.

From a clinical point of view, the current ESC/ERS guidelines recommend a comprehensive assessment of patients with PAH, since no single assessed parameter provides adequate prognostic information.^11^ As a result, the ESC/ERS guidelines proposed a multiparametric approach that was later validated by several registries such as the Swedish Pulmonary Arterial Hypertension Register (SPAHR),^12^ COMPERA,^13^ and FPHR.^14^ While validated, the multiparametric approach is still subject for further refinement, as demonstrated by concordance statistics based on data from several studies, ranging from 0.56 to 0.76.^153233^ Hence, integration of prognostic biomarkers to the multiparametric strategy, providing further prognostic information may be a possible approach to improve the current multiparametric-based risk assessment. In the present study, we found that vWF was associated with survival and predicted mortality with a sensitivity of 81.25% and specificity of 93.75%, after adjusting for age, sex, and combining with the ESC/ERS risk score. Nonetheless, emphasis should be put on that the predictive ability of mortality was based on a logistic regression model, in which some information is lost compared to a COX-regression ditto. Concordant with our results, high levels of vWF were shown to be associated with worse survival and risk of death; the latter being independent of demographics, baseline haemodynamics, or PAH therapy.^34^ Intriguingly, elevated levels of plasma vWF have been associated with histological evidence of endothelial cell injury in PAH,^35^ and dysfunctional endothelial cells release large amounts of structurally deranged vWF with decreased biological activity.^36^ In patients with PH, the deranged properties of circulating vWF includes diminished levels of large multimeric vWF and an increase of low molecular weight polymers.^36^ Considering these findings, our results of diminished levels of ADAMTS13 in PAH patients may seem paradoxical. However, previous explanations have been suggested, including abnormal proteolytic activity, consumption of large multimeric vWF, or unprocessed production of abnormal vWF by endothelial cells.^3637^ Thus, we believe that the underlying process of low levels of multimeric vWF may be multifactorial, where ADAMTS13 may not be the only contributor of the decrease in multimeric vWF. Given that the activity of vWF (i) encompasses the interaction between platelets and endothelial cells^8^ and (ii) is related to its cleaving protease ADAMTS13,^27^ it is possible that the interplay of the vWF-ADAMTS13 axis has a role in endothelial dysfunction in PAH (Figure 3). Thus, further studies are needed to better define such relation in PAH, by for instance assessing the size and biological activity of vWF in PAH.

We consider the use of PEA for measurement of plasma proteins, the invasive haemodynamic assessment, the incident PAH population, and the internal validation as strengths in the present study. In addition, samples were collected at different times and during non-fasting conditions, reflecting the putative future practical implementation by being independent of fasting, and diurnal variations. Despite these strengths, several limitations should be acknowledged. First, our study is single centered with low number of patients, and as a result, the scarcity of events limited the liberty to adjust for additional variables including medications and comorbidities. However, the present study is a unique initial screening evaluation, and several future studies are needed to confirm and validate our results. Next, although we performed internal validation, the method is not free from bias, emphasizing the necessity of applying external validation in future studies. In the present work, the study population encompassed SSc-PAH and patientes with connective tissue disease associated PAH. Only cases where the pulmonary fibrosis component was negligible were included. As such, the development of a more “mixed disease” at a later stage cannot be ruled out. Although the age of the present PAH cohort may be considered somewhat high, it is concordant with the age reported in the SPAHR and COMPERA registries.^3839^ Lastly, although PEA provides high sensitivity and specificity,^17^ the AU level output is based on the Ct values, thus limiting the applicability of, for instance, stated thresholds across other studies. Nonetheless, our work provides important and novel insight into the potential applicability of ADAMTS13 and vWF.

## Conclusion

In the present study, we have identified plasma ADAMTS13 as a differentiator of PAH from the other dyspnoea-related disease groups including CTEPH, HFpEF-PH, HFrEF-PH, HF-non-PH, as well as from healthy controls. Using a multivariable logistic regression model, ADAMTS13 levels adjusted for age and sex discriminated PAH patients from the other dyspnoea-related disease groups with an AUC of 0.91, a sensitivity of 87.5% and a specificity of 78.4%. Furthermore, plasma vWF is related to survival and prognosis in patients with PAH. Baseline plasma vWF, adjusted for age, sex, and combined with ESC/ERS risk stratification score predicted mortality in patients with PAH with an AUC of 0.94, a sensitivity of 81.25% and specificity of 93.75%. Altogether, plasma ADAMTS13 may be a candidate biomarker for early diagnosis and referral of PAH, and vWF as a potential prognostic biomarker with possible additional value to current multiparametric risk stratification instruments. Larger studies are needed to confirm the diagnostic accuracy of ADAMTS13 in PAH and investigate the potential additional value of vWF to the multiparametric ESC/ERS risk assessment instrument.

## Supplemental Material

sj-pdf-1-pul-10.1177_20458940211041500 - Supplemental material for Plasma ADAMTS13 and von Willebrand factor in diagnosis and prediction of prognosis in pulmonary arterial hypertension Click here for additional data file. Supplemental material, sj-pdf-1-pul-10.1177_20458940211041500 for Plasma ADAMTS13 and von Willebrand factor in diagnosis and prediction of prognosis in pulmonary arterial hypertension by Abdulla Ahmed, Salaheldin Ahmed and Göran Rådegran in Pulmonary Circulation

sj-pdf-2-pul-10.1177_20458940211041500 - Supplemental material for Plasma ADAMTS13 and von Willebrand factor in diagnosis and prediction of prognosis in pulmonary arterial hypertension Click here for additional data file. Supplemental material, sj-pdf-2-pul-10.1177_20458940211041500 for Plasma ADAMTS13 and von Willebrand factor in diagnosis and prediction of prognosis in pulmonary arterial hypertension by Abdulla Ahmed, Salaheldin Ahmed and Göran Rådegran in Pulmonary Circulation

sj-pdf-3-pul-10.1177_20458940211041500 - Supplemental material for Plasma ADAMTS13 and von Willebrand factor in diagnosis and prediction of prognosis in pulmonary arterial hypertension Click here for additional data file. Supplemental material, sj-pdf-3-pul-10.1177_20458940211041500 for Plasma ADAMTS13 and von Willebrand factor in diagnosis and prediction of prognosis in pulmonary arterial hypertension by Abdulla Ahmed, Salaheldin Ahmed and Göran Rådegran in Pulmonary Circulation

sj-pdf-4-pul-10.1177_20458940211041500 - Supplemental material for Plasma ADAMTS13 and von Willebrand factor in diagnosis and prediction of prognosis in pulmonary arterial hypertension Click here for additional data file. Supplemental material, sj-pdf-4-pul-10.1177_20458940211041500 for Plasma ADAMTS13 and von Willebrand factor in diagnosis and prediction of prognosis in pulmonary arterial hypertension by Abdulla Ahmed, Salaheldin Ahmed and Göran Rådegran in Pulmonary Circulation

sj-pdf-5-pul-10.1177_20458940211041500 - Supplemental material for Plasma ADAMTS13 and von Willebrand factor in diagnosis and prediction of prognosis in pulmonary arterial hypertension Click here for additional data file. Supplemental material, sj-pdf-5-pul-10.1177_20458940211041500 for Plasma ADAMTS13 and von Willebrand factor in diagnosis and prediction of prognosis in pulmonary arterial hypertension by Abdulla Ahmed, Salaheldin Ahmed and Göran Rådegran in Pulmonary Circulation
