# Supplementary material for: Plasma ADAMTS13 and von Willebrand factor in diagnosis and prediction of prognosis in pulmonary arterial hypertension
Source: Pulm Circ. 2021 Sep 30;11(4):20458940211041500. doi: 10.1177/20458940211041500 (PMC8488531; doi:10.1177/20458940211041500)
Supplement: sj-pdf-3-pul-10.1177_20458940211041500 - Supplemental material for Plasma ADAMTS13 and von Willebrand factor in diagnosis and prediction of prognosis in pulmonary arterial hypertension [file sj-pdf-3-pul-10.1177_20458940211041500.pdf]

STROBE Statement—checklist of items that should be included in reports of observational studies

	Item No.	Recommendation	Page No.	Relevant text from manuscript
**Title and abstract**	1	(*a*) Indicate the study's design with a commonly used term in the title or the abstract		Page 4, mentioned in the methods section.
		(*b*) Provide in the abstract an informative and balanced summary of what was done and what was found		Page 1.
**Introduction**				
Background/rationale	2	Explain the scientific background and rationale for the investigation being reported	Pages 2-3.	
Objectives	3	State specific objectives, including any prespecified hypotheses	Page 3.	
**Methods**				
Study design	4	Present key elements of study design early in the paper	Page 4.	
Setting	5	Describe the setting, locations, and relevant dates, including periods of recruitment, exposure, follow-up, and data collection	Page 4.	
Participants	6	(*a*) *Cohort study*—Give the eligibility criteria, and the sources and methods of selection of participants. Describe methods of follow-up<br>*Case-control study*—Give the eligibility criteria, and the sources and methods of case ascertainment and control selection. Give the rationale for the choice of cases and controls<br>*Cross-sectional study*—Give the eligibility criteria, and the sources and methods of selection of participants	Pages 4-7.	
		(*b*) *Cohort study*—For matched studies, give matching criteria and number of exposed and unexposed<br>*Case-control study*—For matched studies, give matching criteria and the number of controls per case		
Variables	7	Clearly define all outcomes, exposures, predictors, potential confounders, and effect modifiers. Give diagnostic criteria, if applicable	Pages 7-9.	
Data sources/ measurement	8*	For each variable of interest, give sources of data and details of methods of assessment (measurement). Describe comparability of assessment methods if there is more than one group	Pages 4-9.	*Data on controls and patients are stated in detail.*
Bias	9	Describe any efforts to address potential sources of bias	Pages 14-15.	
Study size	10	Explain how the study size was arrived at	Page 4.	

Continued on next page

Quantitative variables	11	Explain how quantitative variables were handled in the analyses. If applicable, describe which groupings were chosen and why	Pages 7-9.
Statistical methods	12	(*a*) Describe all statistical methods, including those used to control for confounding	Pages 7-9.
		(*b*) Describe any methods used to examine subgroups and interactions	Pages 8, 9.
		(*c*) Explain how missing data were addressed	Page 4.
		(*d*) *Cohort study*—If applicable, explain how loss to follow-up was addressed<br>*Case-control study*—If applicable, explain how matching of cases and controls was addressed<br>*Cross-sectional study*—If applicable, describe analytical methods taking account of sampling strategy	Pages 8-10.
		(*e*) Describe any sensitivity analyses	-
**Results**			
Participants	13*	(a) Report numbers of individuals at each stage of study—eg numbers potentially eligible, examined for eligibility, confirmed eligible, included in the study, completing follow-up, and analysed	Pages 4, 10-12.
		(b) Give reasons for non-participation at each stage	Pages 4, 8.
		(c) Consider use of a flow diagram	-
Descriptive data	14*	(a) Give characteristics of study participants (eg demographic, clinical, social) and information on exposures and potential confounders	Pages 10, 17.
		(b) Indicate number of participants with missing data for each variable of interest	Pages 17-20, 22.
		(c) *Cohort study*—Summarise follow-up time (eg, average and total amount)	Page 10.
Outcome data	15*	*Cohort study*—Report numbers of outcome events or summary measures over time	*Pages 10, 17.*
		Case-control study—Report numbers in each exposure category, or summary measures of exposure	
		Cross-sectional study—Report numbers of outcome events or summary measures	
Main results	16	(*a*) Give unadjusted estimates and, if applicable, confounder-adjusted estimates and their precision (eg, 95% confidence interval). Make clear which confounders were adjusted for and why they were included	Pages 11, 12, 21, 24.
		(*b*) Report category boundaries when continuous variables were categorized	Page 15, 16.
		(*c*) If relevant, consider translating estimates of relative risk into absolute risk for a meaningful time period	Not applicable.

Continued on next page

Other analyses	17	Report other analyses done—eg analyses of subgroups and interactions, and sensitivity analyses	Pages 10, 11, 14.
**Discussion**			
Key results	18	Summarise key results with reference to study objectives	Page 13.
Limitations	19	Discuss limitations of the study, taking into account sources of potential bias or imprecision. Discuss both direction and magnitude of any potential bias	Pages 15, 16.
Interpretation	20	Give a cautious overall interpretation of results considering objectives, limitations, multiplicity of analyses, results from similar studies, and other relevant evidence	Pages 1, 14-16.
Generalisability	21	Discuss the generalisability (external validity) of the study results	Page 15, 16.
**Other information**			
Funding	22	Give the source of funding and the role of the funders for the present study and, if applicable, for the original study on which the present article is based	Provided in a separate document, page 2.

*Give information separately for cases and controls in case-control studies and, if applicable, for exposed and unexposed groups in cohort and cross-sectional studies.

Note: An Explanation and Elaboration article discusses each checklist item and gives methodological background and published examples of transparent reporting. The STROBE checklist is best used in conjunction with this article (freely available on the Web sites of PLoS Medicine at http://www.plosmedicine.org/, Annals of Internal Medicine at http://www.annals.org/, and Epidemiology at http://www.epidem.com/). Information on the STROBE Initiative is available at www.strobe-statement.org.
